# Renoprotective Effect of the Shen-Yan-Fang-Shuai Formula by Inhibiting TNF-*α*/NF-*κ*B Signaling Pathway in Diabetic Rats

**DOI:** 10.1155/2017/4319057

**Published:** 2017-06-21

**Authors:** Jie Lv, Zhen Wang, Ying Wang, Weiwei Sun, Jingwei Zhou, Mengdi Wang, Wei Jing Liu, Yaoxian Wang

**Affiliations:** ^1^Dongzhimen Hospital Affiliated to Beijing University of Chinese Medicine, Renal Research Institution, Beijing University of Chinese Medicine, Key Laboratory of Chinese Internal Medicine of Ministry of Education and Beijing, Dongzhimen Hospital Affiliated to Beijing University of Chinese Medicine, Beijing 100700, China; ^2^Zhanjiang Key Laboratory of Prevention and Management of Chronic Kidney Disease, Guangdong Medical University, Zhanjiang, Guangdong 524001, China

## Abstract

Diabetic kidney disease (DKD) is the leading cause of end-stage kidney disease, and satisfactory therapeutic strategies have not yet been established. The Shen-Yan-Fang-Shuai Formula (SYFSF) is a traditional Chinese formula composed of Astragali radix, *Radixangelicae sinensis*, *Rheum officinale* Baill, and four other herbs. It has been widely used as an effective treatment for DKD patients in China. However, little is known about the molecular mechanisms underlying SYFSF's renoprotection. In this study, we compared the protective effect of SYFSF to irbesartan on the histology and renal cells in type 2 DKD rat model and high-glucose (HG) cultured mesangial cells, respectively. We found that SYFSF could significantly decrease urinary albumin, cholesterol, and triglyceride. And a decrease in serum creatinine was also found in SYFSF-treated group compared with irbesartan-treated rats. In addition, SYFSF inhibited the interstitial expansion and glomerulosclerosis in diabetic rats. Notably, SYFSF markedly downregulated the expression of MCP-1, TGF-*β*1, collagen IV, and fibronectin in diabetic rat models and HG-induced mesangial cell models. The renoprotection was closely associated with a reduced expression of TNF-*α* and phosphorylated NF-*κ*Bp65. Our study suggests that SYFSF may ameliorate diabetic kidney injury. The observed renoprotection is probably attributable to an inhibition of inflammatory response and extracellular matrix (ECM) accumulation mediated by TNF-*α*/NF-*κ*Bp65 signaling pathway.

## 1. Introduction

Diabetic kidney disease (DKD) is one of the major microvascular complications of diabetes. It affects around 30% of diabetic patients, making it a leading cause of end-stage renal failure (ESRD) in the Western world [1]. Numerous studies have highlighted the critical role of inflammation in the pathogenesis and progression of DKD [2]. The inflammation is characterized by renal monocyte infiltration in kidney tissue, high expression of proinflammatory cytokines, cell adhesion molecules, and chemokines. The inflammation has a strong relationship with the progression of DKD as indicated by proteinuria, GFR decline, and interstitial fibrosis [3, 4]. Many studies suggest that anti-inflammatory interventions may slow the progression of diabetic kidney disease. The medicines targeting inflammatory molecules such as emapticap pegol, CCR2 antagonist CCX140-B, and baricitinib are under exploration, and many questions remain undetermined [5]. The medicine targeting the renin-angiotensin system (RAS) such as ACEI and ARB is the most validated clinical strategy for slowing DKD progression. The renoprotective effects of RAS inhibitors are probably mostly due to their hemodynamic effects, blood pressure control, and inflammation control [6]. However, the side effects of RAS such as dry cough, hypotension, hyperkalemia, angioedema, and reduction in GFR limit their use [7]. Therefore, the development of new therapeutic agents that prevent and effectively attenuate the DKD progression is most important.

Traditional Chinese medicine (TCM) is widely used for diabetes and its complications in China [8]. Shen-Yan-Fang-Shuai-Formula (SYFSF) is based on the “ZhengJia” theory in traditional Chinese medicine and is widely used in the clinic. Its composition includes Astragali radix, *Radix angelicae sinensis*, *Rheum officinale* Baill, and four other herbs. Cumulative evidence suggests that SYFSF constituents that include emodin and flavonoids have a beneficial role in slowing the progressive renal disease [9]. The mechanisms include anti-inflammation and inhibition of fibrosis. This formula showed antifibrosis and urinary albumin reduction effects in UUO rat model in our previous experiment [10]. However, little is known about the underlying protective mechanisms of SYFSF on DKD.

Nuclear factor-kappa B (NF-*κ*B) is an ubiquitous and well-known transcription factor responsible for regulating the expressions of genes involved in inflammatory pathways such as proinflammatory cytokines, chemokines, and adhesion molecules. Tumor necrosis factor-alpha (TNF-*α*)/NF-*κ*B signaling has been recognized as one of the key pathways in the development of inflammation in many kidney diseases including DKD. It is well established that TNF-*α* activates NF-*κ*B by binding to TNF receptor 1 (TNFR1) and initiates the transcription of a wide variety of target genes. Overexpression of transforming growth factor-*β*1 (TGF-*β*1) promotes extracellular matrix (ECM) production and renal fibrosis [11]. In this study, we investigated the anti-inflammatory and renal protective effects of SYFSF on type 2 DKD rat model.

## 2. Material and Methods

### 2.1. Herbal Formation and Components

SYFSF was extracted from seven natural herbs: Astragali radix (Huang Qi), *Rheum officinale* Baill (Da Huang), *Radix angelicae sinensis* (Dang Gui), sargassum (Hai Zao), Carapax Trionycis (Bie Jia), Concha Ostreae (Mu Li), and *Radix rehmanniae preparata* (Shu Di). The herbs were boiled into decoction and the final concentration was extracted into 1 g/ml. The raw materials of herbs were bought from Tongrentang Company which is well recognized in China for its high quality-control standards. Quality control and the final extraction were performed according to established guidelines in the Pharmacopoeia of The People's Republic of China, 2010 [12]. The irbesartan was purchased from Sanofi (Hangzhou, Zhejiang, China). The final concentration was 0.1 mg/ml in ddH_2_O.

### 2.2. Animals and Experimental Design

60 male Wistar rats (6–8 weeks old, 180–200 g) were purchased from the Beijing Viltariver LLC. The rats were housed in an air-conditioned room at 22–24°C and humidity of 65–69% and were subjected to a 12-hour light/dark cycle with food and water ad libitum. Experimental procedures were approved by the Ethics Committee of Beijing University of TCM and performed in accordance with The National Academies Guiding Principles for the Care and Use of Laboratory Animals, 8th edition.

After one week adaptation, the rats were randomly divided into normal control group (*n* = 10) and DKD group (*n* = 47). The DKD model was induced according to an established protocol [13]. In order to induce type 2 diabetic rats, the DKD model rats were first treated with uninephrectomy to induce hyperfiltration and hyperperfusion and then developed by high-fat diet (67.5% standard fodder, 20% sucrose, 10% lard oil, and 2.5% cholesterol) for 4 weeks. In addition, the DKD rats were treated with a single intraperitoneal injection of 1% streptozotocin (30 mg/kg, i.p.), while the normal control rats received a standard rat chow and an equivalent dose of citrate buffer.

The rats with tail-vein fasting blood glucose > 16.7 mmol/l measured by One Touch UltraII glucometer (Johnson, USA) in two consecutive measurements 72 hours after STZ injection were then randomly divided into 3 subgroups: the DKD group treated with saline water 3 ml/d (*n* = 9), DKD + S group treated with SYFSF in the dose of 11.4 g/kg/d (*n* = 10), and DKD + I group treated with irbesartan in the dose of 35 mg/kg/d (*n* = 10). All drugs were administered via intragastric gavage once per day for 8 weeks.

The 24-hour urine was collected by individual metabolic cages at the 8th week. The quantification of urinary albumin level was measured by Bradford assay according to the manufacturer's instructions (Bradford kit, Nanjing Jiancheng Bioengineering Institute, China). Rats were sacrificed and aortic blood was collected without anticoagulant and centrifuged at 3000*g*/min, 4°C for 15 min at the end of the 8th week. Serum cholesterol, creatinine, and triglyceride levels were measured using the Olympus AU5800 Hematology analyzer (Olympus, Japan).

### 2.3. Cell Culture

Rat renal mesangial cell line (HBZY-1) was purchased from China Infrastructure of Cell Line Resource. Cells were cultured in DMEM medium (containing 5.6 mM D-(+)-glucose, 10% FBS, 100 mg/ml streptomycin, 100 mg/ml penicillin) at 37°C. The 7th to 10th passages of the cells were used for experiments. The cells were divided into four groups cultured in medium with different glucose concentrations: (1) 5.6 mM glucose DMEM medium; (2) 30 mM glucose DMEM medium; (3) 30 mM glucose DMEM medium with serum from rats fed with irbesartan (the final concentration was 10%); and (4) 30 mM glucose DMEM medium with serum from rats fed with SYFSF (the final concentration was 10%).

### 2.4. Histology and Immunohistochemistry

Kidneys and cells were fixed in 4% polyformaldehyde for 24 h and then embedded in paraffin, and 4 *μ*m sections were cut. Sections were stained with HE, periodic acid-Schiff (PAS), and Masson. Glomerulosclerosis was defined as the percentage of extracellular matrix (ECM) deposition and mesangial expansion; all these sores were evaluated at 400 × power in a blinded manner for 50 full-size glomeruli. The tubulointerstitial damage including tubular dilation and atrophy, cast formation, interstitial mononuclear cell, and ECM accumulation (interstitial volume) was scored in 20 cortical fields each group at 200 × power. The semiquantitative scoring was as follows: 0 = almost normal; 1 = less than 10%; 2 = 10%~25%; 3 = 25%~50%; and 4 = more than 50%. The averages of the glomerulosclerosis and tubulointerstitial injury scores were calculated from the total evaluated glomeruli or tubulointerstitial lesions in each section.

A heat-based antigen retrieval method was applied to conduct immunohistochemistry. The primary antibodies against TNF-*α* (ab4418), TGF-*β*1 (ab169771), collagen IV (ab6586), and NF-*κ*Bp65 (ab7970) were purchased from Abcam. And the secondary antibody and DBA including Dako REAL™ EnVision™ Detection System peroxidase/DAB+ were purchased from Dako. After primary and secondary antibody staining, sections were developed with diaminobenzidine to produce a brown product. The sections were then counterstained with hematoxylin. The images with 30 seperate glomeruli were collected by Anymicro DSSTM system, and the degree of TNF-*α*, NF-*κ*Bp65, TGF-*β*1, collagen IV, and fibronectin deposition was rated as grades 1 (none), 2 (minor), 3 (moderate), 4 (severe), and 5 (most severe). The data were expressed as fold change of control. All counts were performed on blinded slides.

### 2.5. Western Blot Analysis

Proteins for western blot analysis were extracted from the renal cortex by lysing with NETN150 (0.5% NP-40, Tris PH 8.0 50 mM, NaCl 150 mM). The proteins were quantified by Bradford, and the samples were separated by SDS PAGE. Gels were transferred to the nitrocellulose membrane (Axygen, Union City, CA) and then blocked with 5% nonfat milk for 1 hour at room temperature, the membrane was incubated with primary antibodies overnight at 4°C, followed by horseradish peroxidase- (HRP-) linked secondary antibody. The antibodies against TGF-*β*1 (ab169771) and NF-*κ*Bp65 (ab7970) were purchased from Abcam, and phospho-NF-*κ*Bp65 (Ser536) was bought from Cell Signaling. After usage of Immobilon Western Chemiluminescent HRP Substrate kit (Millipore Corporation, Billerica, MA), the band was quantified with ImageJ (National Institutes of Health, Bethesda, MD, USA).

### 2.6. Statistical Analysis

All of the statistical tests were performed using SPSS 16.0. Data are expressed as the means ± SEM. Multiple-group comparisons were performed using ANOVA followed by Bonferroni or Dunnett's post hoc tests. *P* < 0.05 was considered to be statistically significant.

## 3. Results

### 3.1. SYFSF Decreased Urinary Albumin, Serum Cholesterol, and Triglyceride Levels in DKD Rats

**Figure 1:**
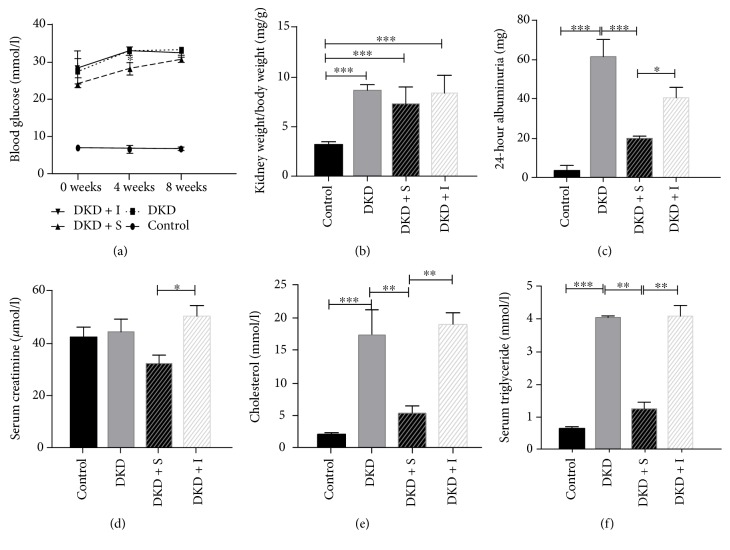
SYFSF attenuated renal injury in DKD rats. (a) Blood glucose was significantly increased after uninephrectomy, STZ-injection, and high-fat-diet induction. The glucose level in the SYFSF group was lower compared with that in the DKD and irbesartan groups. (b) Compared with the normal rats, the diabetic rats developed significant emaciation. (c) The 24-hour urinary protein in the DKD group increased markedly which was significantly suppressed by SYFSF treatment. (d) The levels of serum creatinine between the SYFSF group and the irbesartan group had statistical significance. (e, f) Serum cholesterol and triglyceride levels obviously increased in the diabetic groups, and SYFSF obviously decreased the levels of cholesterol and triglyceride. DKD + S represents the DKD rats treated with SYFSF. DKD + I represents the DKD rats treated with irbesartan. ^∗^*P* < 0.05, ^∗∗^*P* < 0.01, and ^∗∗∗^*P* < 0.001.

After uninephrectomy and STZ injection, rats fed with high-fat diet developed hyperglycemia at the first week (week 0) and maintained at high levels of blood glucose over the 8-week study period. And the serum glucose level in SYFSF group was lower compared with DKD and irbesartan group, especially in the 4th week. However, there was no statistical significance in the 8th week (Figure 1(a)). Compared to the age-matched normal rats, the diabetic rats developed significant weight loss. The kidney weight/body weight was increased in diabetic groups (Figure 1(b)). 24-hour urinary albumin in the DKD group increased markedly which was significantly reduced in the diabetic rats treated with SYFSF (Figure 1(c)). There was no difference of serum creatinine between the DKD model rodents and the normal rats. This finding may be due to the model in the early stage of DKD. Interestingly, compared to the control group, the serum creatinine level in SYFSF group was a little lower than the irbesartan group. And these two groups had statistical significance (Figure 1(d)). Meanwhile, the serum cholesterol and triglyceride level increased in DKD compared to the control group, and SYFSF could decrease the cholesterol and triglyceride levels, while irbesartan had no effects on lipid control (Figures 1(e) and 1(f)).

### 3.2. SYFSF Attenuated the Renal Injury in DKD Rats

**Figure 2:**
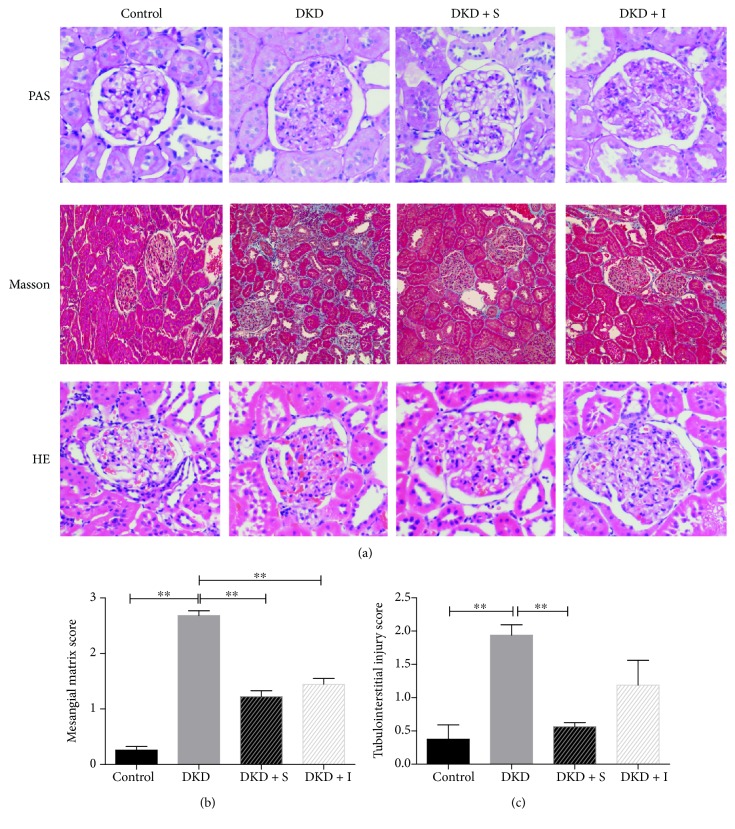
Histological study. (a) HE, PAS, and Masson staining was shown. (b, c) Semiquantification of the mesangial matrix score and the tubular interstitial injury score is presented. DKD + S represents the DKD rats treated with SYFSF. DKD + I represents the DKD rats treated with irbesartan. ^∗∗^*P* < 0.01.

The kidneys from early-stage diabetic rats showed a moderate mesangial matrix expansion, thickening of the glomerular basement membrane, tubular atrophy, and extracellular matrix deposition (Figure 2(a)). Treatment with SYFSF, as well as irbesartan, could ameliorate the mesangial matrix expansion and tubulointerstitial injury (Figures 2(b) and 2(c)).

### 3.3. SYFSF Treatment Inhibited the Inflammation in Mesangial Cells Treated with High Glucose and Kidneys of DKD Rats

**Figure 3:**
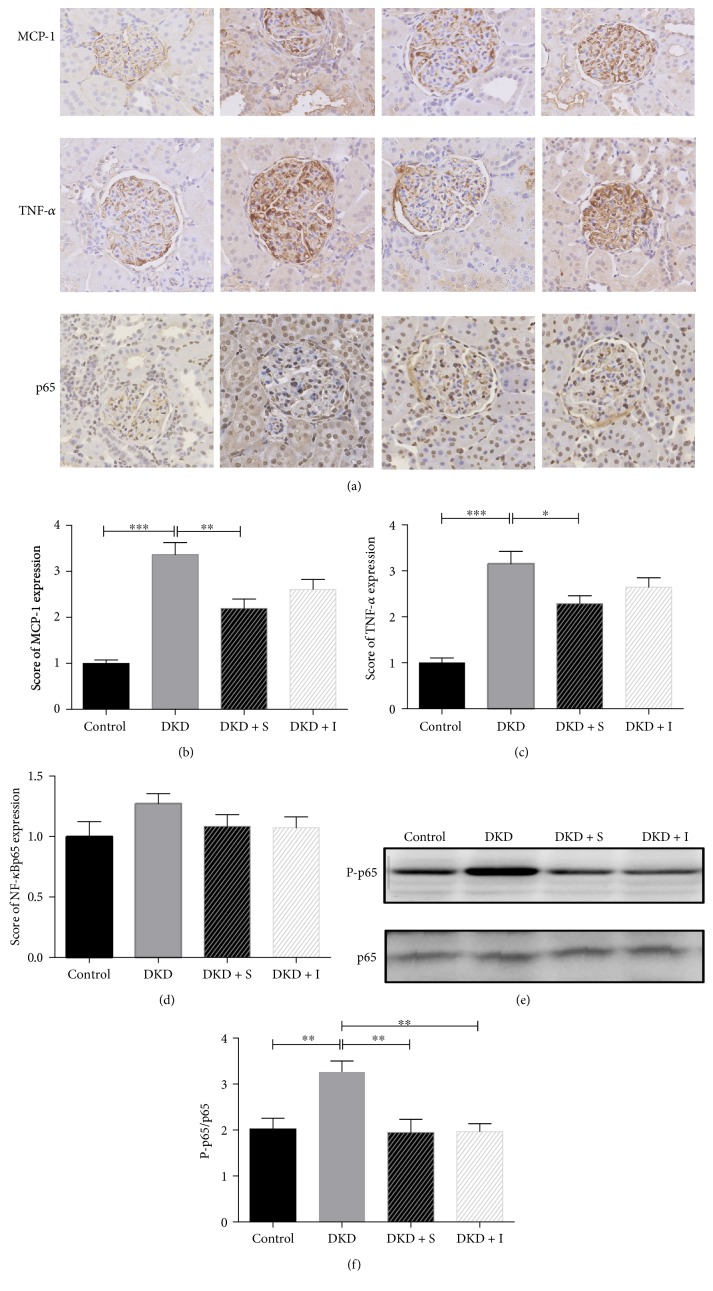
SYFSF serum inhibited renal inflammation in DKD rats. (a) Immunohistochemical staining of MCP-1, TNF-*α*, and NF-*κ*Bp65 is shown. (b, c) The expression of MCP-1, TNF-*α*, and NF-*κ*Bp65 was scored. SYFSF inhibited the expression of MCP-1 and TNF-*α*. (d) There was no significant difference in NF-*κ*Bp65 expression. (e, f) The expression of phosphorylated NF-*κ*Bp65 was obviously increased in the DKD group in western blot. SYFSF and irbesartan had almost similar effects on the inhibition of NF-*κ*Bp65 phosphorylation. DKD + S represents the DKD rats treated with SYFSF. DKD + I represents the DKD rats treated with irbesartan. ^∗^*P* < 0.05, ^∗∗^*P* < 0.01, and ^∗∗∗^*P* < 0.001.

**Figure 4:**
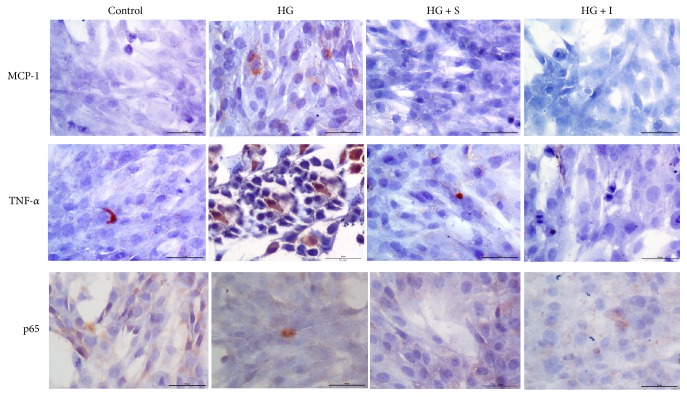
SYFSF inhibited the expression of inflammatory cytokine in high-glucose-treated mesangial cells. High glucose induced upregulation of MCP-1 and TNF-*α* which were suppressed by SYFSF serum in mesangial cells. There was no obvious change of NF-*κ*Bp65 expression. HG + S represents the mesangial cells treated with high-glucose medium and serum from rats treated with SYFSF. HG + I represents the mesangial cells treated with high-glucose medium and serum from rats treated with irbesartan.

Immunohistochemistry revealed that the rats with type 2 diabetes developed moderate renal inflammation, including many macrophage infiltration and a significant upregulation of proinflammatory cytokines including TNF-*α* and monocyte chemoattractant protein-1 (MCP-1) (Figure 3(a)), which was attenuated by treatment with SYFSF. SYFSF showed better anti-inflammatory effect than irbesartan (Figures 3(b) and 3(c)). In addition, the expression of phosphorylated NF-*κ*Bp65 was obviously increased in the DKD group as shown in western blot. SYFSF and irbesartan had almost equal effects on the inhibition of NF-*κ*Bp65 phosphorylation (Figures 3(e) and 3(f)), although there was no marked difference in NF-*κ*Bp65 expression (Figure 3(d)). Similar results were obtained in cultured mesangial cells. The expression of inflammatory cytokines such as MCP-1 or TNF-*α* was increased after exposure to high glucose. SYFSF serum could decrease the expression of MCP-1 and TNF-*α* as shown by immunocytochemistry (Figure 4).

### 3.4. SYFSF Treatment Inhibited Renal Fibrosis in Mesangial Cells Treated with High Glucose and Kidneys of DKD Rats

**Figure 5:**
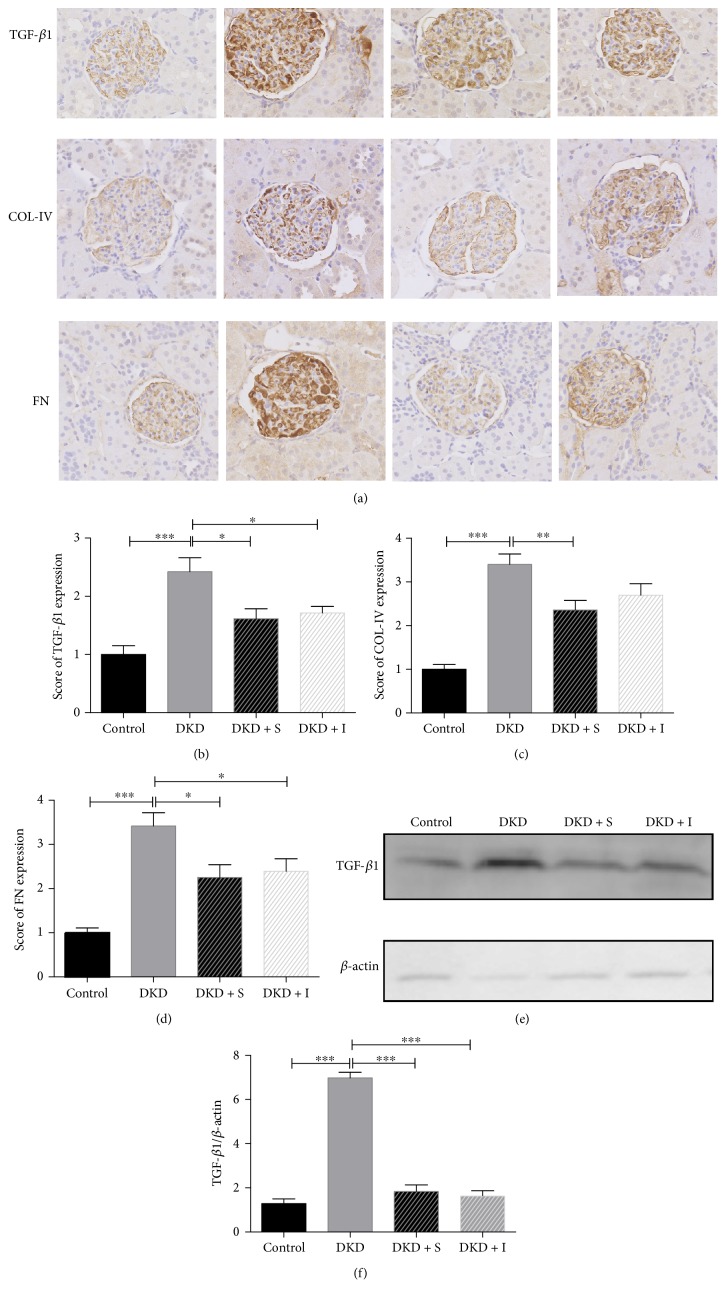
SYFSF inhibited renal fibrosis in DKD rats. (a–d) Immunohistochemical staining of TGF-*β*1, collagen IV, and fibronectin is shown. (e, f) Western blot and quantitative analysis of TGF-*β*1 in the cortex. SYFSF and irbesartan inhibited the expression of TGF-*β*1 in DKD rats. DKD + S represents the DKD rats treated with SYFSF. DKD + I represents the DKD rats treated with irbesartan. ^∗^*P* < 0.05, ^∗∗^*P* < 0.01, and ^∗∗∗^*P* < 0.001.

**Figure 6:**
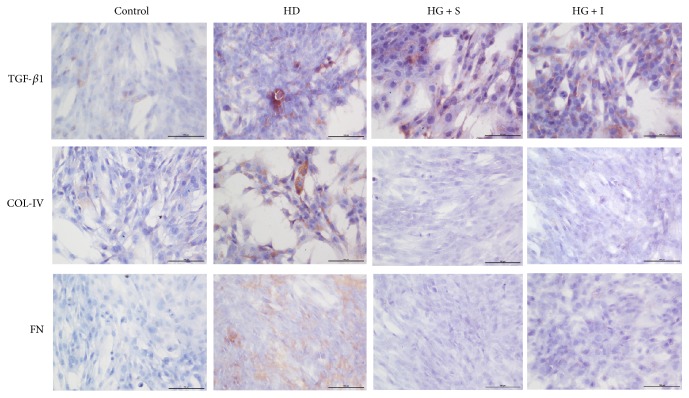
SYFSF serum inhibited fibrotic cytokine expression in high-glucose-treated mesangial cells. SYFSF inhibited the expression of TGF-*β*1, collagen IV, and fibronectin in high-glucose-treated mesangial cells. HG + S represents the mesangial cells treated with high-glucose medium and serum from rats treated with SYFSF. HG + I represents the mesangial cells treated with high-glucose medium and serum from rats treated with irbesartan.

The expression of TGF-*β*1, collagen IV, and fibronectin was increased in the diabetic kidney compared with the control. SYFSF could downregulate the fibrotic cytokines, as shown in immunohistochemistry. Interestingly, SYFSF worked better on the inhibition of collagen IV expression compared with irbesartan (Figure 5). TNF-*α*/NF-*κ*B signaling pathway had been recognized as one of the key pathways in the development of inflammation in DKD. It was well established that TNF-*α* activates NF-*κ*B by binding to TNF receptor 1 (TNFR1) and initiates the transcription of a wide variety of target genes. Overexpression of TGF-*β*1 promoted extracellular matrix production and at the same time inhibits its degradation, resulting in progressive renal fibrosis [14]. We thus investigated the potential mechanisms by which SYFSF treatment attenuated diabetic renal fibrosis and inflammation by examining the TGF-*β*1 expression in protein level. We found that SYFSF and irbesartan significantly suppressed the expression of TGF-*β*1 (Figures 5(e) and 5(f)). Meanwhile, in immunocytochemical study, the similar results were gotten. Exposure to high glucose upregulates the expression of TGF-*β*1, collagen IV, and fibronectin, which were blocked by SYFSF treatment (Figure 6).

## 4. Discussion

The results in the present study provided evidences that the Chinese herbal medicine SYFSF significantly decreased the urinary albumin, serum cholesterol, and triglyceride levels, as well as alleviated the renal lesions in a rat model with type 2 diabetes. The therapeutic effect of SYFSF was associated with the inhibition of TNF-*α*/NF-*κ*B pathway mediated inflammatory response and ECM accumulation. And SYFSF likely played a better role in decreasing urinary albumin, inflammation, and dyslipidemia than irbesartan.

In this study, we successfully developed an experimental animal model with early-stage diabetic kidney disease by uninephrectomy, high-fat diet, and low-dose intraperitoneal injection of streptozotocin. Compared to normal rats, the blood glucose, urinary albumin, and serum cholesterol levels were significantly increased, while the body weight was markedly decreased in DKD animals throughout this experiment. The histological study showed that the excessive mesangial matrix deposition and tubular atrophy, as well as an accumulation of inflammatory cells, were developed in the early stage of DKD rats. For the 8-week treatment, SYFSF could decrease the urinary albumin level, serum cholesterol, and triglyceride levels in comparison to irbesartan. Interestingly, there was no significant difference of serum creatinine between the normal control and DKD model groups. The expression of fibrosis marker increased in immunohistochemistry, which may be attributed to the glomerular hyperfiltration occurred in the early stage of DKD. The increased creatinine level post irbesartan treatment may be related to its side effects when the emaciation temporarily exacerbated the blood volume deficiency. The results suggest that SYFSF are much safer than irbesartan, especially when some dangerous factors occur during kidney injury.

It has been suggested that there is glomerular infiltration of monocytes/macrophages in early-stage DKD patients, accompanied by mild and moderate glomerulosclerosis. In addition, tubulointerstitial injury triggers mononuclear cell infiltration as an inflammatory response that is strongly related to disease progression [15]. In the present study, our findings were consistent with this claim. Meanwhile, SYFSF and irbesartan could inhibit these histological change and mesangial cell injuries. And SYFSF tends to decrease the tubulointerstitial injury score compared with irbesartan. Inhibition of NF-*κ*B-driven renal inflammation may be one important mechanism associated with the renoprotective effects of SYFSF on DKD. NF-*κ*B is a transcription factor broadly expressed in tissues, which is the key molecule in inflammation reaction in DKD [16]. Many proinflammatory cytokines such as TNF-*α* can activate the NF-*κ*B signaling pathway to aggravate the renal inflammatory response resulting in the progression of DKD [17]. And the activation of NF-*κ*B will promote the hyperplasia of mesangial cells and matrix accumulation [18] which will lead to sclerosis and fibrosis. Although there is no big change of NF-*κ*Bp65 expression, the phosphorylated NF-*κ*Bp65 obviously increased in the DKD model group. Interestingly, SYFSF could significantly decrease the phosphorylation of NF-*κ*Bp65.

More important, we also found that SYFSF offered a greater effect on lipid and urinary albumin control than on irbesartan. Some studies have shown that irbesartan can be a benefit to lipid metabolism [19]. However, this action was not found in the present experiment. A number of studies have shown that DKD is a chronic inflammatory disease accompanied with different degrees of lipid disorders, and the glomerulosclerosis seems similar to the atherosclerosis [20, 21]. It is suggested that the low-density lipoprotein may increase the expression of TNF-*α* in mesangial cells, while the inflammatory cytokines contribute to lipid-mediated renal damage [22]. Some studies have also shown that the inflammation will induce lipid accumulation which contributed to podocyte injury and accelerate the progression of DKD [23]. Therefore, the renoprotective effect of SYFSF is at least partially attributed to breaking the vicious circle between inflammatory response and dyslipidemia by inhibiting the TNF-*α*/NF-*κ*B pathway.

SYFSF is composed of Astragali radix (Huang Qi), *Rheum officinale* Baill (Da Huang), *Radix angelicae sinensis* (Dang Gui), sargassum (Hai Zao), Carapax Trionycis (Bie Jia), Concha Ostreae (Mu Li), and *Radix rehmanniae preparata* (Shu Di). The main active metabolites in SYFSF probably include astragaloside IV [24], ferulic acid [25], emodin [26], angelica [27], and astragalus polysaccharide (APS) [28, 29], which have showed anti-inflammation, antioxidant, and controlling lipid effects in many studies, respectively. Therefore, SYFSF likely works through polypharmacology and network pharmacology to produce an overall systemic effect. Moreover, SYFSF did not cause side effects in our study (data not shown). And all these herbs have been safely used in clinical practice in China for thousands of years, indicating the good security and practicability.

**Figure 7:**
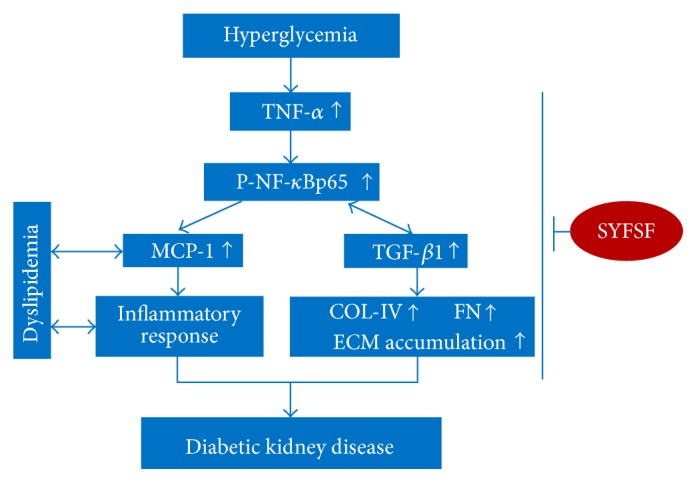
Schematic representation of the mechanism underlying SYFSF's renoprotection which is likely associated with an inhibition of TNF-*α*/NF-*κ*Bp65-mediated signaling pathway. SYFSF probably decreases the expression of TNF-*α* to prevent the phosphorylation of NF-*κ*Bp65 induced by high glucose and then alleviates the inflammatory response and ECM accumulation and normalizes the dyslipidemia.

In conclusion, the present study demonstrates that SYFSF exerts renoprotective effects in a rat model with type 2 diabetes and mesangial cell model after exposure to high glucose. The renal protective effect may be associated with the inhibition of TNF-*α*/NF-*κ*B-dependent renal inflammation and dyslipidemia, as well as TGF-*β*1-mediated renal fibrosis (Figure 7).
